# Anti-inflammatory and anxiolytic activities of *Euphorbia hirta* extract in neonatal asthmatic rats

**DOI:** 10.1186/s13568-018-0707-z

**Published:** 2018-11-01

**Authors:** Mingyue Xia, Ling Liu, Ruiqin Qiu, Mingli Li, Wei Huang, Gaowei Ren, Jinghui Zhang

**Affiliations:** Department of Pediatrics, The First Hospital of Qinhuangdao, No. 258 of Seaport Cultural Road, Qinhuangdao, 066000 China

**Keywords:** Asthma, *Euphorbia hirta*, Caspase-3, Inflammation, Neonatal rats

## Abstract

The current study evaluated the anti-inflammatory and anxiolytic activities of *Euphorbia hirta* extract in neonatal asthmatic rats. Rats were assigned to the following groups: group I, sham (normal rats); group II, control (asthmatic rats); group III, *E. hirta* extract (100 µg/100 µl) and group IV, *E. hirta* extract (200 µg/100 µl). We performed a phytoscreening analysis of *E. hirta* extract. Inflammatory cell counts in the bronchoalveolar lavage fluid, levels of anti-inflammatory and antioxidant markers, apoptosis, and a histopathological analysis were carried out. An open field test determined anxiolytic activity, an elevated plus maze, a hole board test, and a cross test. The presence of 9,12,15-octadecatrien-1-ol, pentadecylic acid, ethyl linoleate, 1,2,3-trihydroxy benzene, gamma-tocopherol, 5-hydroxymethyl-2-furancarboxaldehyde, myristic acid, 7,10-octadecadienoic acid methyl ester, phytol, ethyl palmitate, and squalene in *E. hirta* extract was noted. Following treatment with *E. hirta* extract, total leukocytes, eosinophils, tumor necrosis factor-α (TNF-α), interleukin (IL-6), and lipid peroxidation were reduced, whereas antioxidant levels were increased. The mRNA expression levels of TNF-α, inducible nitric oxide synthase, IL-6, cyclooxygenase-2, caspase-3, p53, nerve growth factor precursor, and Bax were reduced, whereas that of Bcl-2 was increased. Apoptosis and caspase-3 protein expression were significantly reduced. Treatment of rats with *E. hirta* extract significantly reduced inflammation and eosinophil infiltration in the lungs. Taken together, these results led us to conclude that *E. hirta* extract has anti-inflammatory and anxiolytic effects on neonatal asthmatic rats with inflammation.

## Introduction

Plants and natural-derived medicines have attracted the attention of several researchers in China and India. Natural-derived medicines account for more than 20% of the drugs prescribed in developing countries (Rates 2001), and more than 70% of people use traditional natural-derived medicines for their health needs (Food and Agriculture Organization 2004). Nair et al. (2005) identified several plant parts that participate in numerous pharmacological activities. *Euphorbia hirta* is a familiar plant in the traditional medicine system and belongs to the family Euphorbiaceae (Kumar et al. 2010). Several researchers have reported that extracts of *E. hirta* perform various pharmacological functions, acting as an anxiolytic, sedative, anti-inflammatory, analgesic, and antipyretic agent. Indeed, it can be used to treat several conditions, such as hay asthma, worm infestations, bronchial infections, kidney stones, and bowel disease (Sharma et al. 2007; Salles et al. 1999).

Asthma is a well-known chronic disease of the lungs that affects the airways (Martinez 2007). Primary symptoms of asthma include bronchospasm, airflow obstruction, chest tightness, coughing, wheezing, and shortness of breath (Han et al. 2017). Asthma cannot be cured permanently; only the symptoms can be ameliorated by medications (Han et al. 2017). Avoidance of smoking, aspirin, and pets are the most efficient way to ameliorate asthmatic symptoms, and drugs are recommended based on symptom severity (Kelly et al. 1988). Bronchodilators are widely prescribed for the temporary relief from asthmatic symptoms (Shichinohe et al. 1996). Several studies have reported the antioxidant, anticancer, and anti-inflammatory activities of *E. hirta* extract (Sharma et al. 2014). Thus, the current study evaluated the anti-inflammatory and anxiolytic effects of *E. hirta* extract on neonatal asthmatic rats.

## Materials and methods

### Rats

Male Sprague–Dawley neonatal rats (6–10 g) were obtained from the animal house of The First Hospital of Qinhuangdao, No. 258 of Seaport Cultural Road, Qinhuangdao 066000, China and divided into four groups. They were allowed standard access to food and water with a standard 12-h light/dark period. All animals were maintained on adaptive feeding before the experiments. All the animal experiments were approved by ethics committee of The First Hospital of Qinhuangdao, No. 258 of Seaport Cultural Road, Qinhuangdao 066000, China.

### Induction of asthma and animal groups

Experimental asthma was induced in neonatal rats according to the previously described method (Feng et al. 2014). The following groups were used:

Group I: Sham (normal rats);
Group II: Control (asthmatic rats);
Group III: *Euphorbia hirta* extract (asthmatic rats + 100 µg/100 µl); and
Group IV: *Euphorbia hirta* extract (asthmatic rats + 200 µg/100 µl)

The dose was given for 5 consecutive weeks via the oral route.

### Preparation of *E. hirta* leaf extract

*Euphorbia hirta* leaves were collected from South China Agricultural University, China. Fresh leaves of *E. hirta* were harvested, washed in tap water, and air-dried for 7 days. Next, leaves were ground into a fine powder using an electric grinder and stored in an air-tight bottle. *Euphorbia hirta* powder (500 g) was extracted in 500 ml of ethanol (95%) using a Soxhlet apparatus for 60 min. Finally, the extract was converted into solid form using a rotary evaporator (Sharma et al. 2014).

### Qualitative analysis of *E. hirta* extract

Qualitative analysis of lemon peel extract was performed using high-performance liquid chromatography (HPLC). To determine the presence of a bioactive substance in *E. hirta* extract, HPLC was performed on a liquid chromatography system (Agilent 1200, Germany). Compounds were monitored on a C18 HPLC column. Ten microliters of sample were injected, and the column was operated at room temperature. The flow rate, which was adjusted to 0.5 ml/min, consisted of a mobile phase containing acetonitrile (100%) and acetic acid (0.5%). Peaks were identified by comparing known standards and retention times (Jiang and Tu 2009).

### Collection of blood and preparation of lung tissue homogenate

At the end of treatment, rats were anesthetized by intraperitoneal administration of ketamine (100 mg/kg) and xylazine (10 mg/kg). Rats were killed by decapitation, and blood was collected. Lung tissue was surgically removed, and tissues were cut and homogenized in Tris–HCl buffer (pH 7.4, 50 mM) at 10,000 rpm for 10 min. Tissue homogenate was centrifuged, and the supernatant was collected for further experiments. All homogenate and supernatant preparations were performed at 4 °C.

### Determination of inflammatory cell counts

Inflammatory cell counts in the bronchoalveolar lavage fluid (BALF) were measured according to the previously described method (Feng et al. 2014).

### Determination of inflammatory and antioxidant markers

Serum tumor necrosis factor-α (TNF-α) and interleukin-6 (IL-6) levels were determined (Tavakkol Afshari et al. 2005). The nitric oxide (NO) level is expressed in mM (Shaheen et al. 2016). The mRNA expression levels of TNF-α, IL-6, cyclooxygenase-2 (COX-2), and inducible nitric oxide synthase (iNOS) were measured according to the previously described method (Bernal et al. 2005). Primers specific to the above genes are shown in Table 1. Lipid peroxidation was determined according to malondialdehyde (MDA) content (Jordão et al. 2004). Reactive oxygen species (ROS) were determined using the dichlorofluorescein assay (Arutyunyan et al. 2016). The reduced glutathione (GSH) level was measured by determining the final product at 405 nm (ErdenInal et al. 2003). Superoxide dismutase (SOD), catalase, glutathione peroxidase (Gpx), and acetyl-cholinesterase (AChE) enzyme activities were measured spectrophotometrically (Jordão et al. 2004).

**Table 1:** List of RT-PCR primers used for the amplification of TNF-α, IL-6, COX-2, iNOS, caspase-3, proNGF, p53, bax and bcl-2

S. no	Gene name	Forward	Reverse
1	TNF-α	5′-CCCAGACCCTCACACTCAGAT-3′	5′-TTGTCCCTTGAAGAGAACCTG-3′
2	IL-6	5′-AAGTTTCTCTCCGCAAGATACTTCCAGCCA-3′	5′-AGGCAAATTTCCTGGTTATATCCA GTTT-3′
3	COX-2	5′-CCATGTCAAAACCGTGGTGAATG-3′	5′-ATGGGAGTTGGGCAGTCATCAG-3′
4	iNOS	5′-CTCCATGACTCTCAGCACAGAG-3′	5′-GCACCGAAGATATCCTCATGAT-3′
5	Caspase-3	5′-TTAATAAAGGTATCCATGGAGAACACT-3′	5′-TTAGTGATAAAAATAGAGTTCTTTTGTGAG-3′
6	proNGF	5′-CTTCAGCATTCCCTTGACAC-3′	5′-TGAGCACACACACGCAGGC-3′
7	p53	5′-TAACAGTTCCTGCATGGGCGGC-3′	5′-AGGACAGGCACAAACACGCACC-3′
8	Bcl-2	5′-CACCCCTGG CATCTTCTCCTT-3′	5′-AGCGTCTTCAGAGACAGCCAG-3′
9	Bax	5′-TGG AGCTGCAGAGGATGATTG-3′	5′-GAAGTTGCCGTCAGAAAACATG-3′
10	GAPDH	5′-TCCCTCAAGATTGTCAGCAA-3′	5′-AGATCCACAACGGATACATT-3′

### Determination of apoptosis

Terminal deoxynucleotidyl transferase dUTP nick end labeling (TUNEL) and staining was performed in lung sections as previously described (Sheikh-Hamad et al. 2004). Briefly, lung tissues were excised and perfused in normal saline, and then fixed in 10% neutral formalin (10%) for 24 h. Next, the lung sections were dehydrated with a graded series of alcohol and embedded in paraffin film. Finally, the sections were cut (4–5 µm thick) with a rotary microtome and TUNEL staining was performed as previously described (Fayzullina and Martin 2014). The mRNA expression levels of caspase-3, p53, Bcl-2, Bax, and nerve growth factor precursor (proNGF) were determined according to a previously described method (Bernal et al. 2005). Primers specific to the above genes are shown in Table 1. Protein expression of caspase-3 was analyzed by immunohistochemical staining (Rehg et al. 2012).

### Histopathological analysis

Lung tissue was fixed in 10% neutral formalin and sliced into sections (4–5 µm). Sections were then incubated with hematoxylin and eosin (H&E) and analyzed under a microscope (Ding et al. 2011).

### Determination of anxiolytic activity

Anxiolytic activity was determined using an open field test, an elevated plus maze, a hole board test, and a cross test according to a previous study (Amdadul et al. 2015).

### Statistical analysis

Values are given as the mean ± standard deviation. One-way analysis of variance (ANOVA) was applied, and Tukey’s post hoc test was used for multiple comparisons. A P-value < 0.05 was considered statistically significant.

## Results

The anti-inflammatory and anxiolytic effects of *E. hirta* extract on neonatal asthmatic rats were investigated. The presence of 9,12,15-octadecatrien-1-ol (15.3%), pentadecylic acid (11.6%), ethyl linoleate (8.3%), 1,2,3-trihydroxybenzene (2.2%), gamma-tocopherol (0.92%), 5-hydroxymethyl-2-furancarboxaldehyde (6.3%), myristic acid (1.3%), 7,10-octadecadienoic acid methyl ester (7.1%), phytol (3.2%), ethyl palmitate (6.5%), and squalene (1.2%) in *E. hirta* extract was noted.

**Fig. 1:**
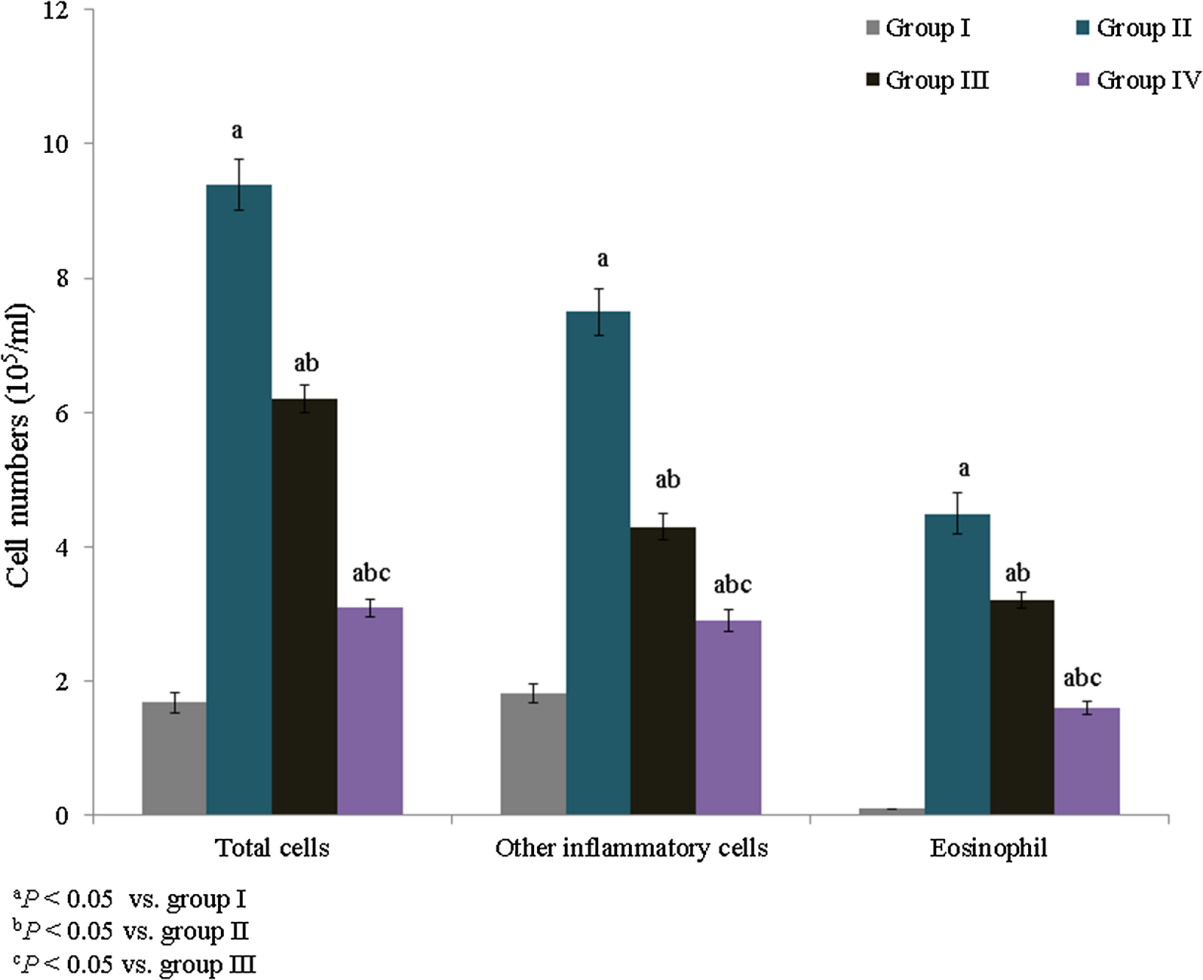
Effect of *E. hirta* extract on inflammatory cell recruitment in the bronchoalveolar lavage fluid (BALF) of neonatal asthmatic rats. Experimental data are given as the mean ± standard error of the mean (SEM)

The total leukocyte content was substantially increased in neonatal asthmatic rats compared to normal rats. Specifically, the total leukocyte content was increased by 459.5% in asthmatic rats, whereas treatment with *E. hirta* extracts significantly reduced the leukocyte content to 34% and 67% in groups III and IV, respectively (Fig. 1, *P *< 0.05). The eosinophil content was substantially increased in asthmatic rats. Treatment with *E. hirta* extracts significantly reduced the eosinophil content to 28.9% and 64.4% in groups III and IV, respectively (Fig. 1, *P *< 0.05). The MDA content was determined as the end-product of lipid peroxidation and was drastically increased in control rats (65.1 nmol/g). Treatment with *E. hirta* extract significantly reduced the MDA content to 23.9% and 46.2% in groups III and IV, respectively (Table 2, *P *< 0.05). The GSH content was drastically reduced in neonatal asthmatic rats (27.5 mg/g), but it was significantly elevated, to 67.6% and 153%, in groups III and IV, respectively (Table 2, *P *< 0.05). SOD, catalase, Gpx, and AChE enzyme activities were substantially reduced in asthmatic rats. However, treatment with *E. hirta* extract significantly increased enzyme activities (Table 2, *P *< 0.05).

**Table 2:** Effect of *E. hirta* extract on lipid peroxidation and antioxidant markers in neonatal asthmatic rats

Oxidative markers	Group I	Group II	Group III	Group IV
MDA (nmol/g)	25.5 ± 1.6	69.1 ± 4.2^a^	52.6 ± 3.1^ab^	37.3 ± 2.4^abc^
GSH (mg/g)	89.2 ± 6.2	27.5 ± 1.8^a^	46.1 ± 3.2^ab^	69.6 ± 5.2^bc^
SOD (U/mg)	7.3 ± 0.54	2.5 ± 0.13^a^	3.5 ± 0.12^ab^	4.9 ± 0.15^abc^
Catalase (U/g)	13.5 ± 1.21	4.2 ± 0.24^a^	6.9 ± 0.23^ab^	10.5 ± 0.8^bc^
Gpx (mg/protein)	0.96 ± 0.008	0.28 ± 0.005^a^	0.47 ± 0.005^ab^	0.77 ± 0.006^bc^
AChE (µmol/min/mg of protein)	10.5 ± 0.73	3.1 ± 0.15^a^	6.3 ± 0.22^ab^	8.9 ± 0.32^bc^

^a^*P* < 0.05 vs. group I

^b^*P* < 0.05 vs. group II

^c^*P* < 0.05 vs. group III

**Fig. 2:**
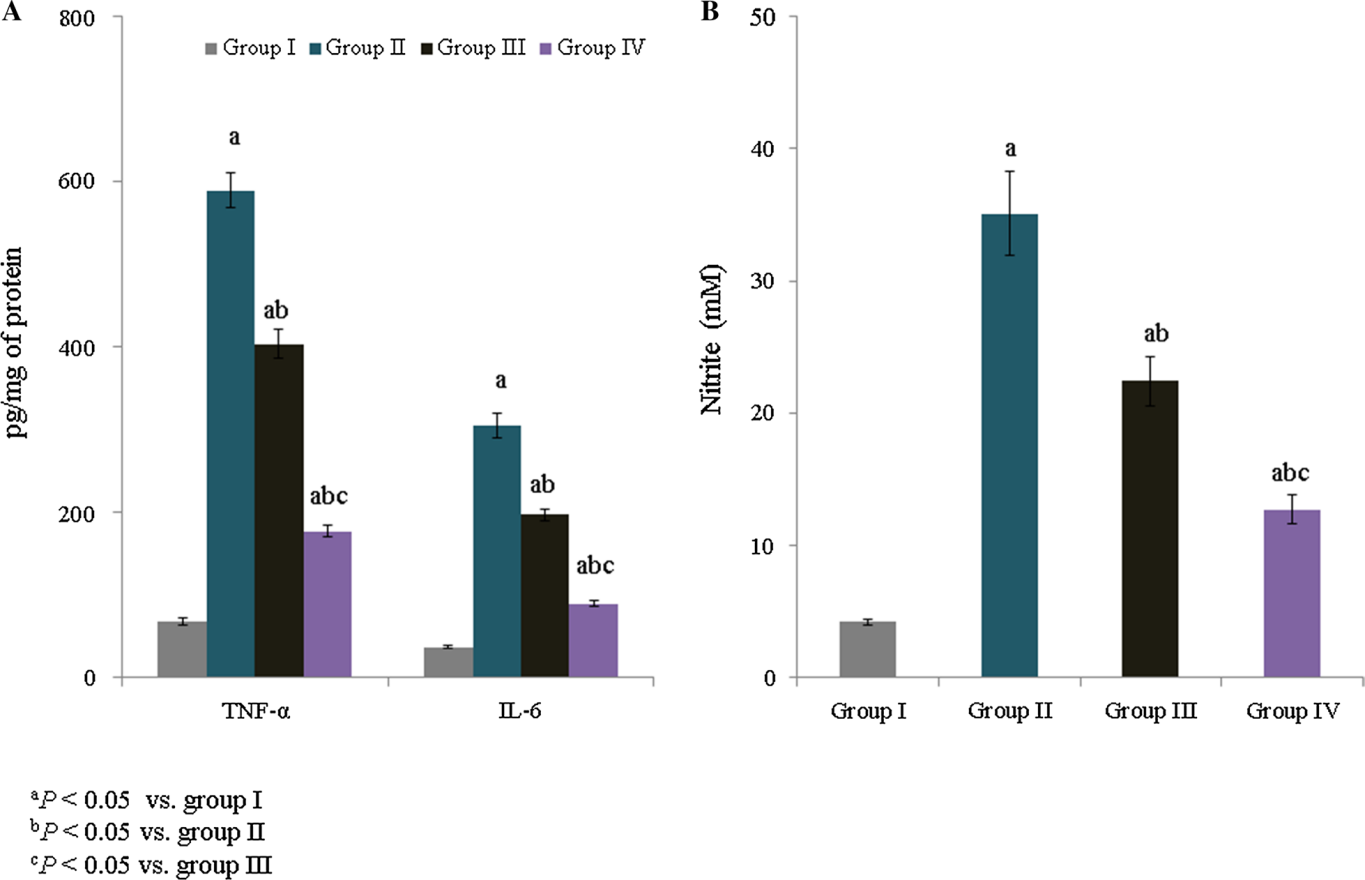
Effect of *E. hirta* extract on tumor necrosis factor alpha (TNF-α), interleukin 6 (IL-6), and nitric oxide (NO) levels in neonatal asthmatic rats. **A** TNF-α and IL-6 levels are expressed as pg/mg of protein. **B** NO is expressed as mM. Experimental data are given as the mean ± SEM

The IL-6 and TNF-α level were measured and are expressed as pg/mg of protein. The IL-6 and TNF-α levels were substantially increased (to more than 100%) in asthmatic rats. However, treatment with *E. hirta* extracts significantly reduced the TNF-α level to 31.5% and 70% in groups III and IV, respectively (Fig. 2A, *P *< 0.05). The IL-6 level was also reduced following supplementation with *E. hirta* extract (Fig. 2A, *P *< 0.05). The NO concentration was substantially increased (to more than 100%) in asthmatic rats. However, treatment with *E. hirta* extracts significantly reduced the NO level, to 36.2% and 63.8%, in groups III and IV, respectively (Fig. 2B, *P *< 0.05).

**Fig. 3:**
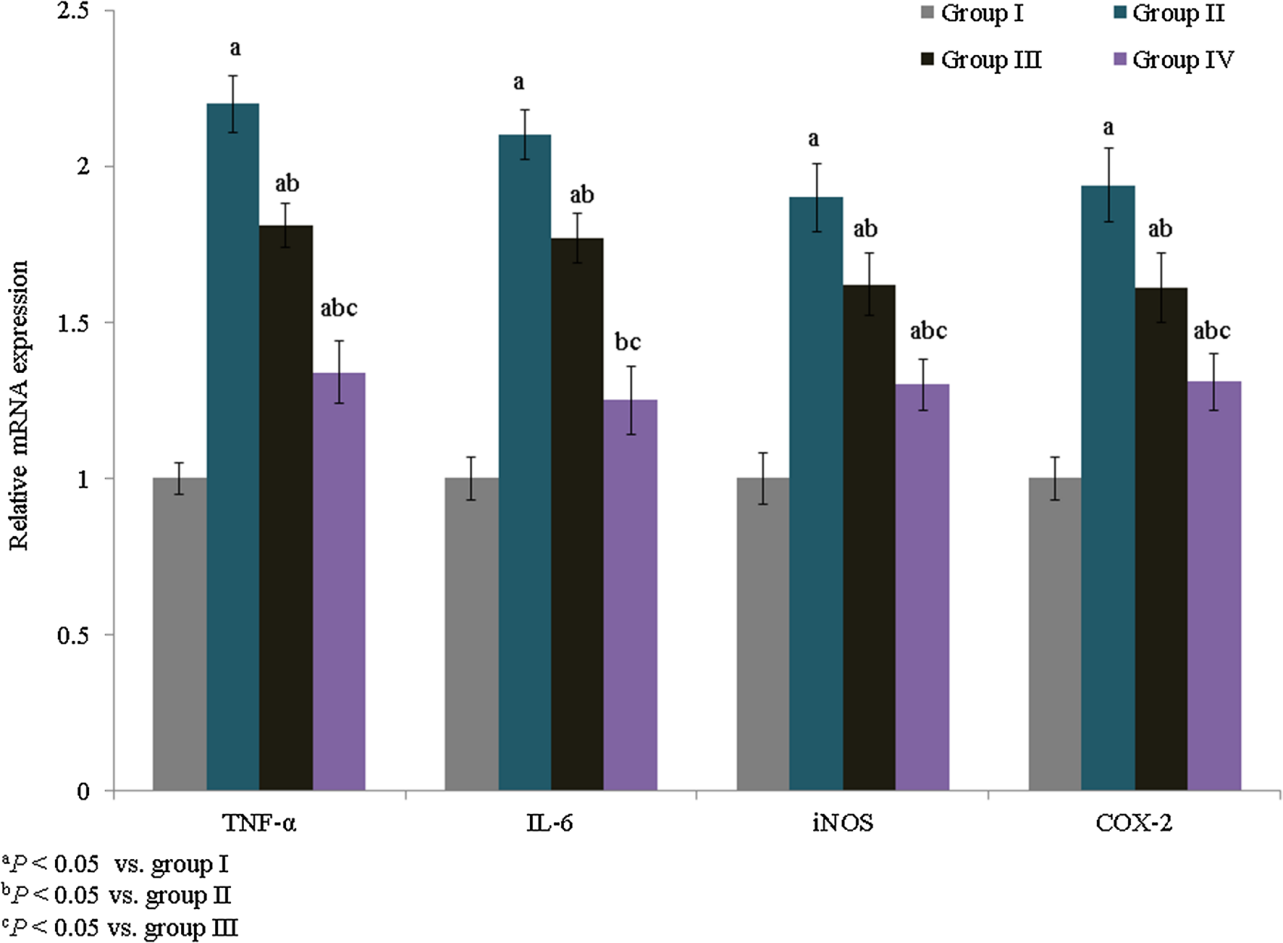
Effect of *E. hirta* extract on the mRNA expression of TNF-α, IL-6, inducible nitric oxide synthase (iNOS), and cyclooxygenase 2 (COX-2) in neonatal asthmatic rats. Experimental data are given as the mean ± SEM

**Fig. 4:**
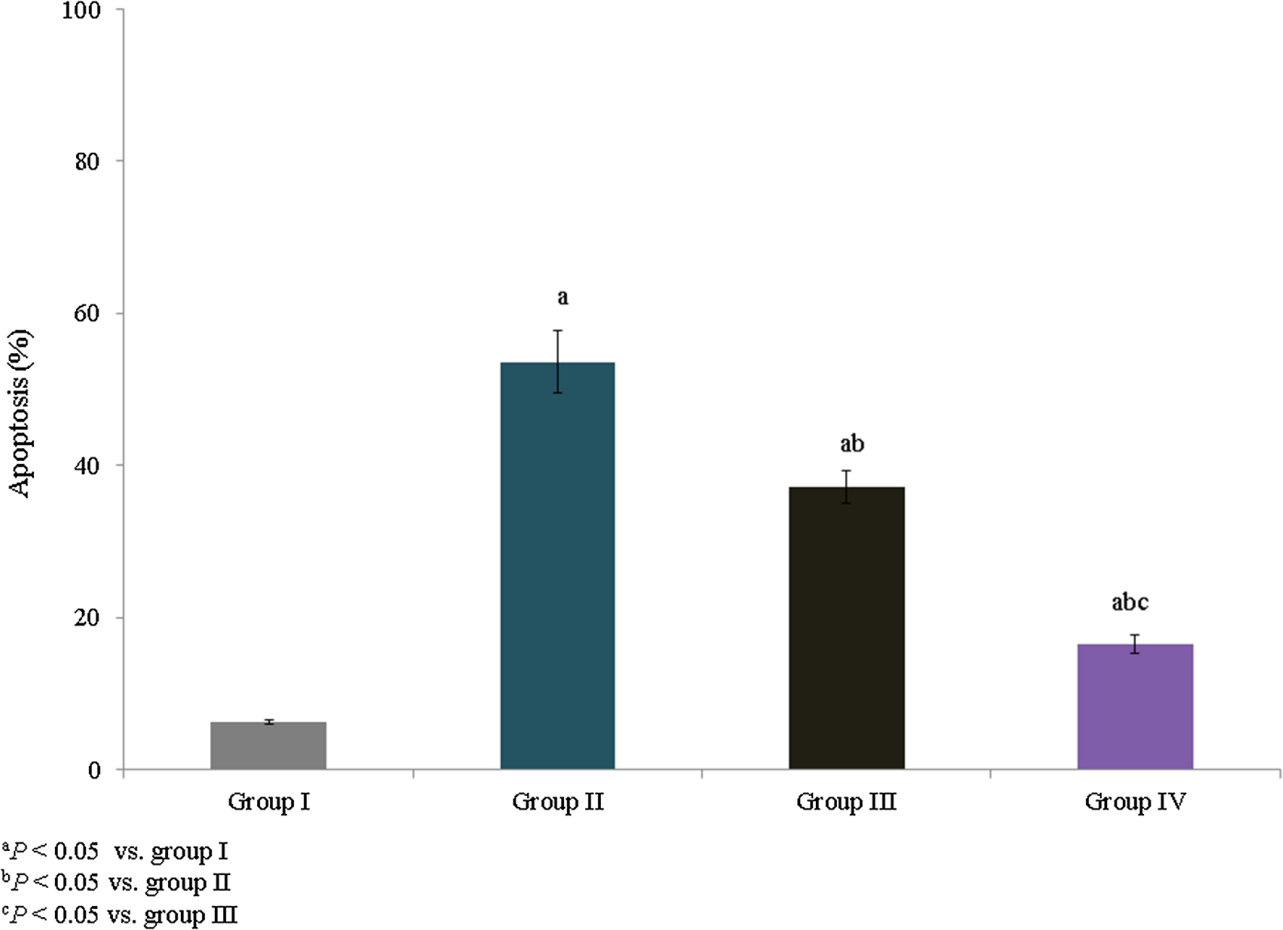
Effect of *E. hirta* extract on apoptosis in neonatal asthmatic rats. Experimental data are given as the mean ± SEM

**Fig. 5:**
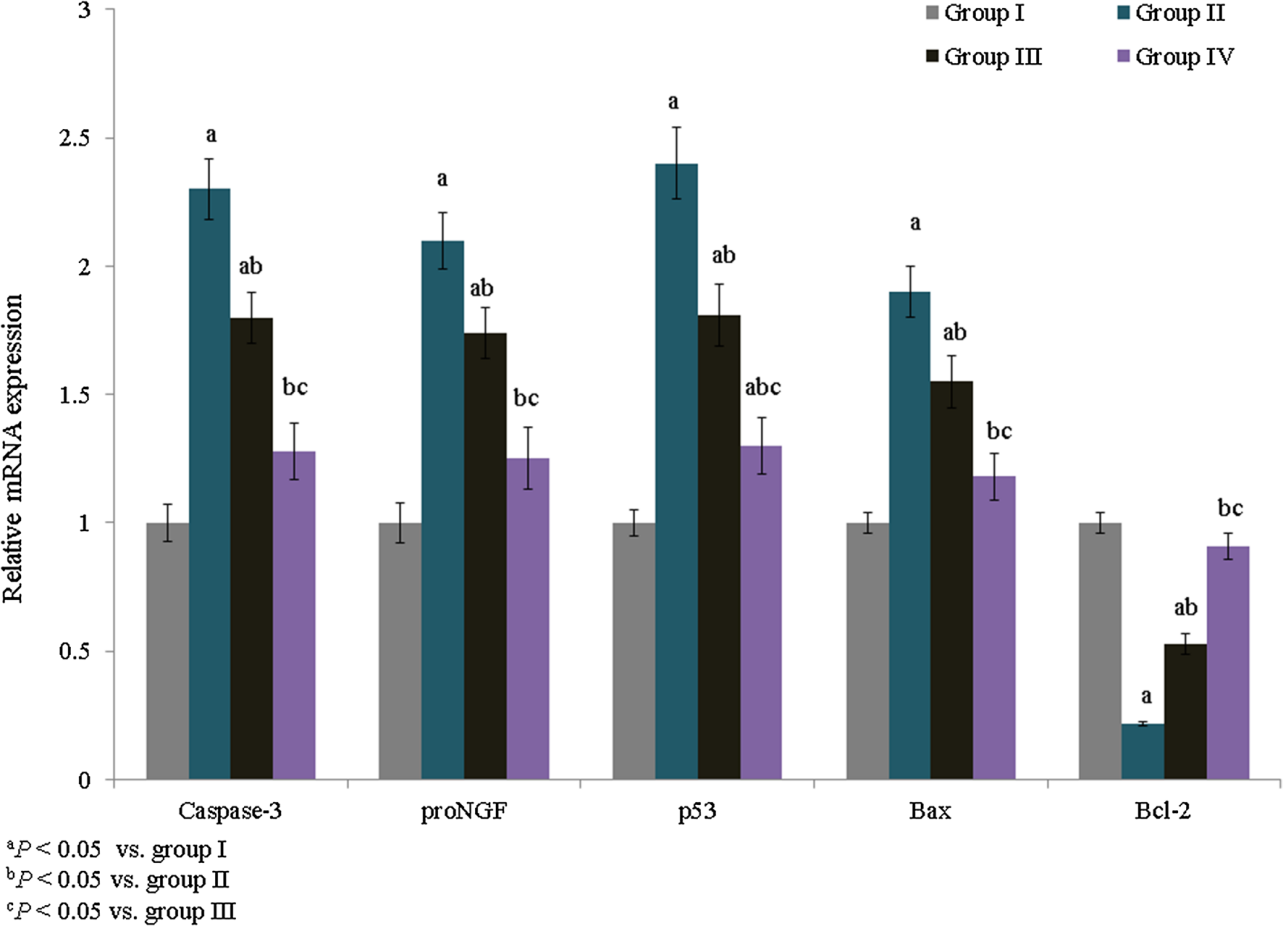
Effect of *E. hirta* extract on the mRNA expression of caspase-3, proNGF, p53, Bax, and Bcl-2 in neonatal asthmatic rats. Experimental data are given as the mean ± SEM

The mRNA expression levels of IL-6, TNF-α, iNOS, and COX-2 were quantified and expressed as fold changes. TNF-α, IL-6, iNOS, and COX-2 mRNA expression was increased by 1.2-, 1.1-, 0.8-, and 0.94-fold, respectively, in group II. However, treatment with *E. hirta* extracts significantly reduced TNF-α, IL-6, iNOS, and COX-2 mRNA expression by more than 0.6-fold in group IV (Fig. 3, *P *< 0.05). The percentage of apoptosis was substantially increased in asthmatic rats (53.6%), whereas treatment with *E. hirta* extracts significantly reduced apoptosis to 30.8% and 69.4% in groups III and IV, respectively (Fig. 4, *P *< 0.05). The mRNA expression levels of inflammatory markers, caspase-3, proNGF, p53, Bax, and Bcl-2, were quantified and expressed as fold changes. The mRNA expression levels of caspase-3, proNGF, p53, and Bax were increased by 1.3-, 1.1-, 1.4-, and 0.9-fold, respectively, in group II, whereas Bcl-2 expression was reduced by 0.78-fold. However, treatment with *E. hirta* extracts significantly reduced the mRNA expression levels of caspase-3, proNGF, p53, and Bax by more than 0.5-fold in group IV (Fig. 5, *P *< 0.05). Bcl-2 mRNA expression was increased by 1.4- and 3.1-fold in groups III and IV, respectively (Fig. 5, *P *< 0.05).

**Fig. 6:**
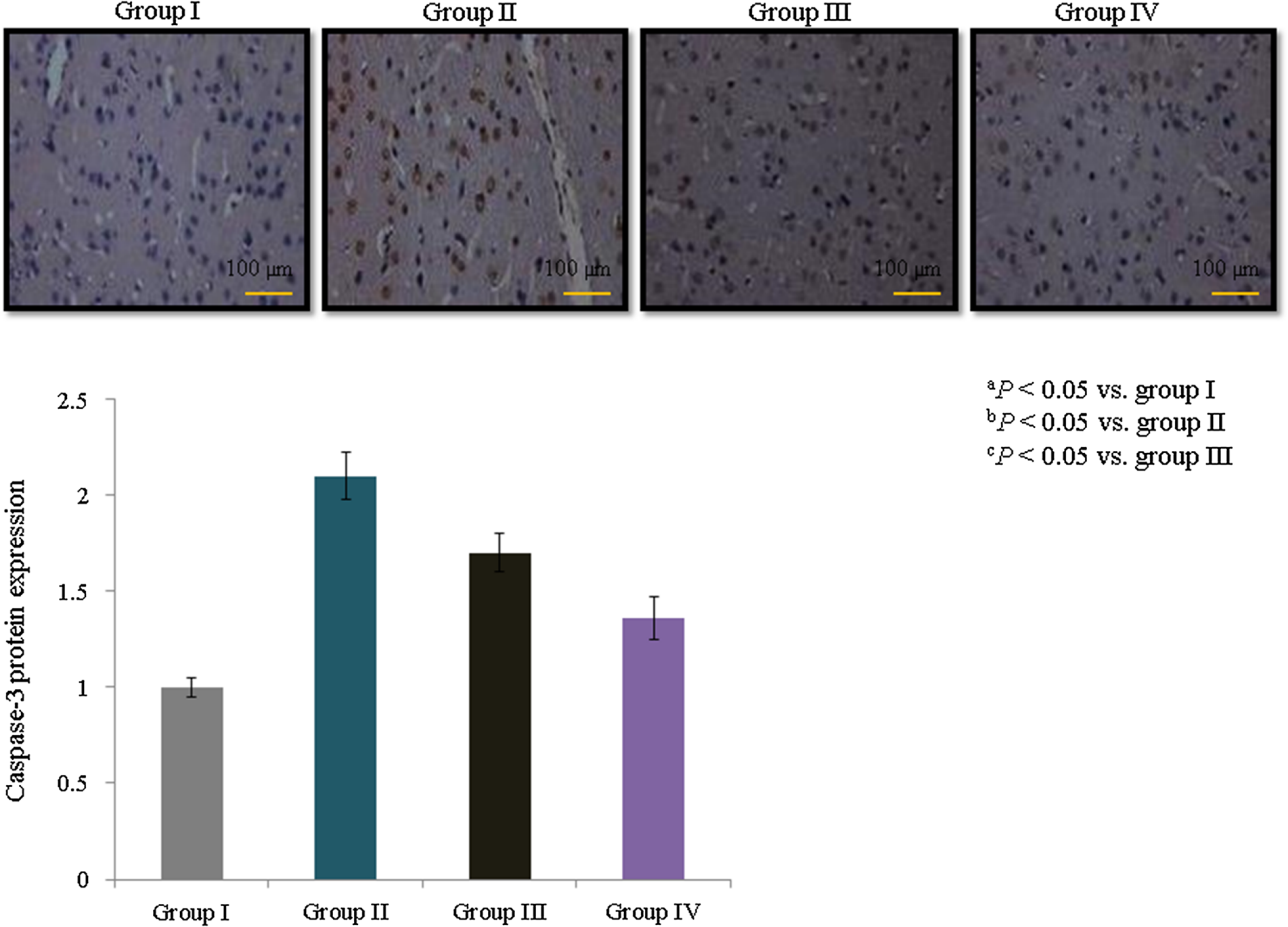
Effect of *E. hirta* extract on caspase-3 protein expression in neonatal asthmatic rats. Experimental data are given as the mean ± SEM

**Fig. 7:**
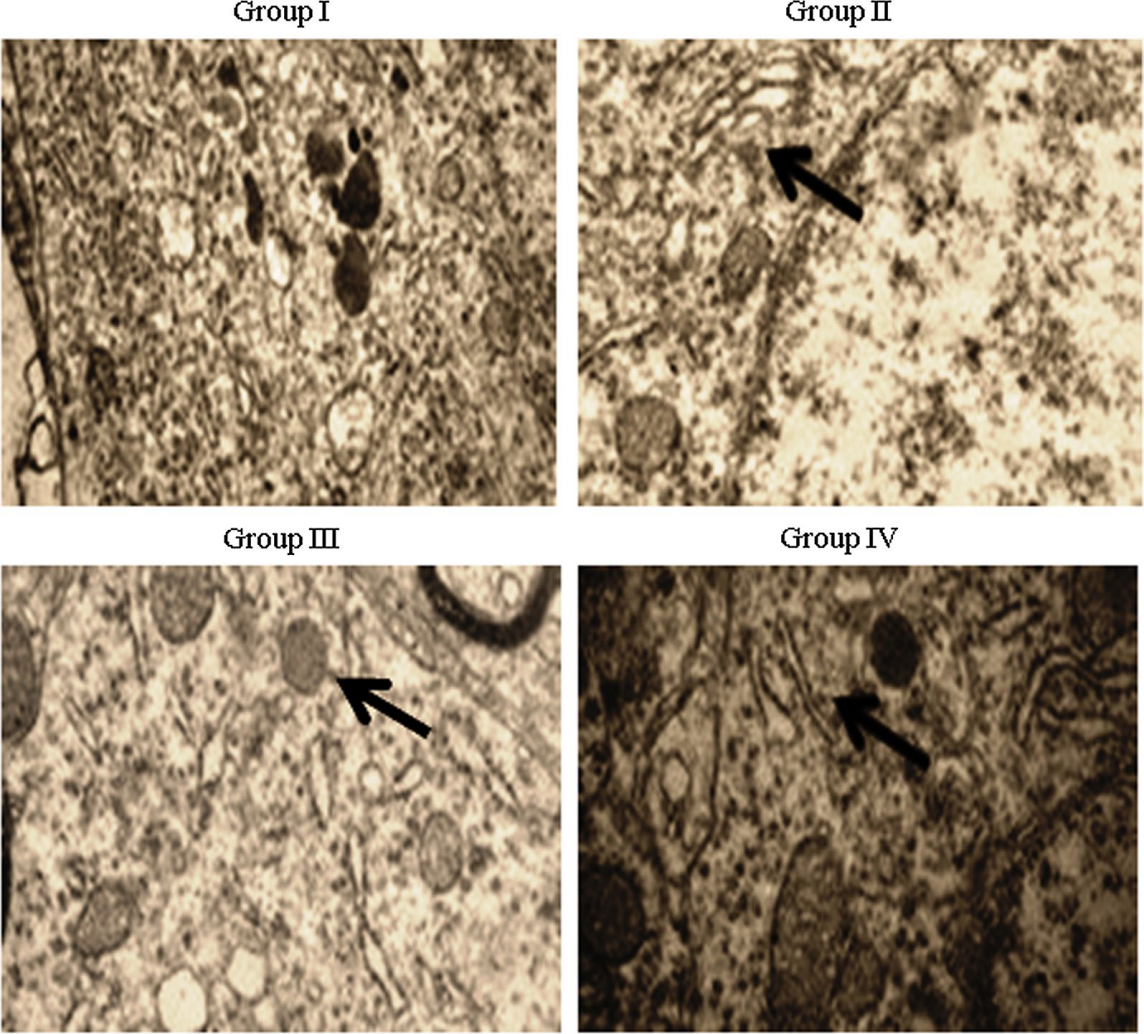
Effect of *E. hirta* extract on the cellular architecture of lungs in neonatal asthmatic rats. Lung sections were stained and evaluated under a microscope. Magnification, ×40 and n = 6

Caspase-3 protein expression was determined immunohistochemically, and its expression increased dramatically (1.1-fold) in asthmatic rats. However, treatment with *E. hirta* extracts significantly reduced caspase-3 protein expression by 19% and 35.2% in groups III and IV, respectively (Fig. 6, *P *< 0.05). Airway and blood vessel narrowing and the accumulation of eosinophils in the lungs of neonatal asthmatic rats were noted. However, treatment with *E. hirta* extracts significantly reduced inflammation and eosinophil infiltration in the lungs (Fig. 7). The open field test was conducted to assess behavior and movement patterns. At higher concentrations, *E. hirta* extract significantly improved the behavior and movement patterns of asthmatic rats compared to their respective controls (Table 3, *P* < 0.05). The elevated plus maze test was performed to assess the anxiolytic activity of *E. hirta* extract. At a higher concentration, *E. hirta* extract exhibited significant anxiolytic activity in asthmatic rats compared to their respective controls (Table 3, *P* < 0.05). At a higher concentration, *E. hirta* extract was associated with significant increases in scores on the hole board and hole cross tests in asthmatic rats compared to their respective controls (Table 3, *P* < 0.05).

**Table 3:** Effect of *E. hirta* extract on the ambulation in open field test, elevated plus maze, hole board and cross test

Groups	Open field test	Elevated plus maze test	Hole board test	Hole cross test
Group I	0	0	0	0
Group II	1.25 ± 0.16^a^	13.42 ± 1.5^a^	8.5 ± 0.71^a^	9.4 ± 0.9^a^
Group III	0.83 ± 0.12^a^	8.2 ± 0.73^ab^	6.2 ± 0.4^a^	6.6 ± 0.7^a^
Group IV	0.24 ± 0.021^abc^	3.1 ± 0.03^abc^	2.1 ± 0.2^abc^	2.2 ± 0.2^abc^

^a^*P* < 0.05 vs. group I

^b^*P* < 0.05 vs. group II

^c^*P* < 0.05 vs. group III

## Discussion

We evaluated the anti-inflammatory and anxiolytic effects of *E. hirta* extract on neonatal asthmatic rats. Several researchers have reported that the extract of *E. hirta* exerts various pharmacological effects, including acting as an anxiolytic, sedative, anti-inflammatory, analgesic, and antipyretic agent. Indeed, it can be used to treat several conditions, such as hay asthma, worm infestations, bronchial disease, kidney stones, and bowel disease (Sharma et al. 2007; Salles et al. 1999). Researchers have reported the anti-inflammatory activity of hydroxymethyl-2-furancarboxaldehyde (Brustugun et al. 2005; Xu et al. 2007). Graff and Pollack (2005) reported the protective effect of hydroxymethyl-2-furancarboxaldehyde against liver injury.

Kagoura et al. (1999) reported the various pharmacological activities of phytol, including its anti-inflammatory effects. Kagoura et al. (1999) also reported the anti-inflammatory and anti-bacterial effects of myristic acid. Wang et al. (2011) reported the antibacterial and antioxidant activities of 9,12,15-octadecatrien-1-ol against *Staphylococcus aureus*. The known activities of these compounds in *E. hirta* confirm our experimental findings. Heo et al. (2006) reported the critical role of phenolic compounds in free radical scavenging, and the antioxidant activity of these phenolic compounds is very crucial for decomposing peroxides and neutralizing free radicals (Kitada et al. 1979).

In this study, the bioactive compounds (phenolic compounds, 9,12,15-octadecatrien-1-ol, squalene and others) accounted for the anti-oxidant activity of *E. hirta* extract. Basma et al. (2011) reported the anti-oxidant activity of the methanolic extract of *E. hirta*. Several researchers have reported that the presence of fatty acids, phytol, and others in *E. hirta* extract accounts for its anti-inflammatory effects (Liu and Huang 2012; Kagoura et al. 1999). Shih et al. (2010) reported that the presence of flavones, glucosides, and tannins contributes to its anti-inflammatory activity through the inhibition of NO. Lefkowitz et al. (1999) reported increased NO generation through the increased production of cytokines, iNOS, and prostaglandins. Researchers have indicated that increased NO production leads to oxidative stress, DNA damage and cell injury (Murphy 1999). Asthma is a well-known chronic airway inflammatory disorder that affects the lungs and causes coughing, wheezing, and chest tightness (Lee et al. 2010). The infiltration of eosinophils, lymphocytes, and mast cells into the airway wall results in mucus hypersecretion and causes allergic inflammation (Bochner and Busse 2004, 2005). Lung histopathological analysis confirmed severe inflammation in asthma-induced rats. However, treatment with *E. hirta* extract significantly attenuated inflammation, reflecting the protective effect of *E. hirta* against asthmatic inflammation. In summary, we conclude that *E. hirta* extract exerts anti-inflammatory and anxiolytic effects against asthmatic inflammation in neonatal rats.

## Abbreviations

TNF-α: tumor necrosis factor-α
IL-6: interleukin
iNOS: inducible nitric oxide synthase
COX-2: cyclooxygenase-2
proNGF: nerve growth factor precursor
HPLC: high-performance liquid chromatography
BALF: bronchoalveolar lavage fluid
NO: nitric oxide
MDA: malondialdehyde
ROS: reactive oxygen species
GSH: reduced glutathione
SOD: superoxide dismutase
Gpx: glutathione peroxidase
AChE: acetyl-cholinesterase
TUNEL: terminal deoxynucleotidyl transferase dUTP nick end labeling

## References

[CR1] Arutyunyan TV, Korystova AF, Kublik LN, Levitman MK, Shaposhnikova VV, Korystov YN (2016). Taxifolin and fucoidin abolish the irradiation-induced increase in the production of reactive oxygen species in rat aorta. Bull Exp Biol Med.

[CR2] Basma AA, Zakaria Z, Latha LY, Sasidharan S (2011). Antioxidant activity and phytochemical screening of the methanol extracts of *Euphorbia hirta* L. Asian Pac J Trop Med.

[CR3] Bernal F, Hartung HP, Kieseier BC (2005). Tissue mRNA expression in the rat of newly described matrix metalloproteinase. Biol Res.

[CR4] Bochner BS, Busse WW (2004). Advances in mechanisms of allergy. J Allergy Clin Immunol.

[CR5] Bochner BS, Busse WW (2005). Allergy and asthma. J Allergy Clin Immunol.

[CR6] Brustugun J, Tonnesen HH, Edge R, Navaratnam S (2005). Formation and reactivity of free radicals in 5-hydroxymethyl-2-furaldehyde—the effect on isoprenaline photostability. J Photochem Photobiol B.

[CR7] Ding Y, Zou J, Li Z, Tian J, Abdelalim S, Du F, She R, Wang D, Tan C, Wang H, Chen W, Lv D, Chang L (2011). Study of histopathological and molecular changes of rat kidney under simulated weightlessness and resistance training protective effect. PLoS ONE.

[CR8] ErdenInal M, Akgün A, Kahraman A (2003). The effects of exogenous glutathione on reduced glutathione level, glutathione peroxidase, and glutathione reductase activities of rats with different ages and gender after whole-body Γ-irradiation. J Am Aging Assoc.

[CR9] Fayzullina S, Martin LJ (2014). Detection and analysis of DNA damage in mouse skeletal muscle in situ using the TUNEL method. J Visualized Exp.

[CR10] Feng D, Chengbin W, Jinyan D, Weiyi Z, Daijun X, Mianyang L (2014). Puerarin attenuates ovalbumin-induced lung inflammation and hemostatic unbalance in rat asthma model. Evid Based Complement Altern Med.

[CR11] Food and Agriculture Organization (2004). Trade in medicinal plants.

[CR12] Graff CL, Pollack GM (2005). Nasal drug administration: potential for targeted central nervous system delivery. J Pharm Sci.

[CR13] Han RT, Kim S, Choi K, Jwa H, Lee J, Kim HY, Kim HJ, Kim HR, Back SK, Na HS (2017). Asthma-like airway inflammation and responses in a rat model of atopic dermatitis induced by neonatal capsaicin treatment. J Asthma Allergy.

[CR14] Heo SJ, Cha SH, Lee KW, Jeon YJ (2006). Antioxidant activities of red algae from Jeju Island. Algae.

[CR15] Huque A, Biswas S, Abdullah-Al-Mamun M, Bhuiyan JR, ur Rashid MH, Jahan A (2015). Analgesic, anti-inflammatory and anxiolytic activity evaluation of methanolic extract of *Solanum surattense* leaf in Swiss Albino mice model. Int J Pharm Clin Res.

[CR16] Jiang Y, Tu PF (2009). Analysis of chemical constituents in *Cistanche* species. J Chromatogr A.

[CR17] Jordão AA, Chiarello PG, Arantes MR, Meirelles MS, Vannucchi H (2004). Effect of an acute dose of ethanol on lipid peroxidation in rats: the action of vitamin E. Food Chem Toxicol.

[CR18] Kagoura M, Matsui C, Morohashi M (1999). Phytol is a novel tumor promoter on ICR mouse skin. Jpn J Cancer Res.

[CR19] Kelly C, Ward C, Stenton CS, Bird G, Hendrick DJ, Walters EH (1988). Number and activity of inflammatory cells in bronchoalveolar lavage fluid in asthma and their relation to airway responsiveness. Thorax.

[CR20] Kitada M, Igaradhi K, Hirose S, Kitagawa H (1979). Inhibition by polyamines of lipid peroxidase formation in rat liver microsomes. Biochem Biophys Res Commun.

[CR21] Kumar S, Malhotra R, Kumar D (2010). *Euphorbia hirta*: its chemistry, traditional and medicinal uses, and pharmacological activities. Pharmacogn Rev.

[CR22] Lee MY, Seo CS, Ha H, Jung D, Lee H, Lee NH, Lee JA, Kim JH, Lee YK, Son JK, Shin HK (2010). Protective effects of *Ulmus davidiana* var. *japonica* against OVA-induced murine asthma model via upregulation of heme oxygenase-1. J Ethnopharmacol.

[CR23] Lefkowitz DL, Gelderman MP, Fuhrmann SR, Graham S, Starnes JD, Lefkowitz SS, Bollen A, Moguilevsky N (1999). Neutrophilic lysozyme-macrophage interactions perpetuate chronic inflammation associated with experimental arthritis. Clin Immunol.

[CR24] Liu CH, Huang HY (2012). Antimicrobial activity of curcumin-loaded myristic acid microemulsions against *Staphylococcus epidermidis*. Chem Pharm Bull.

[CR25] Martinez FD (2007). Genes, environments, development, and asthma: a reappraisal. Eur Respir J.

[CR26] Murphy MP (1999). Nitric oxide, and cell death. Biochim Biophys Acta.

[CR27] Nair R, Kalariya T, Chanda S (2005). Antibacterial activity of some selected Indian medicinal flora. Turk J Biol.

[CR28] Rates SMK (2001). Plants as a source of drugs. Toxicon.

[CR29] Rehg JE, Bush D, Ward JM (2012). The utility of immunohistochemistry for the identification of hematopoietic and lymphoid cells in normal tissues and the interpretation of proliferative and inflammatory lesions of mice and rats. Toxicol Pathol.

[CR30] Salles B, Sattler U, Bozzato C, Calsou P (1999). Repair of oxidative DNA damage in vitro: a tool for screening antioxidative compounds. Food Chem Toxicol.

[CR31] Shaheen TI, El-Naggar MI, Hussein JS, El-Bana M, Emara E, El-Khayat Z, Fouda MMG, Ebaid H, Hebeish A (2016). Antidiabetic assessment; in vivo study of gold and core-shell silver-gold nanoparticles on streptozotocin-induced diabetic rats. Biomed Pharmacother.

[CR32] Sharma JN, Al-Omran A, Parvathy SS (2007). Role of nitric oxide in inflammatory diseases. Inflammopharmacology.

[CR33] Sharma N, Samarakoon KW, Gyawali R, Park YH, Lee SJ, Oh SJ, Lee TH, Jeong DK (2014). Evaluation of the antioxidant, anti-inflammatory, and anticancer activities of *Euphorbia hirta* ethanolic extract. Molecules.

[CR34] Sheikh-Hamad D, Cacini W, Buckley AR, Isaac J, Truong LD, Tsao CC, Kishore BK (2004). Cellular and molecular studies on cisplatin-induced apoptotic cell death in rat kidney. Arch Toxicol.

[CR35] Shichinohe K, Shimizu M, Kurokawa K (1996). Effect of M-711 on experimental asthma in rats. J Vet Med Sci.

[CR36] Shih MF, Cheng YD, Shen CR, Cherng JY (2010). A molecular pharmacology study into the anti-inflammatory actions of *Euphorbia hirta* L. on the LPS-induced RAW 264.7 cells through selective iNOS protein inhibition. J Nat Med.

[CR37] Tavakkol Afshari J, Ghomian N, Shameli A, Shakeri MT, Fahmidehkar MA, Mahajer E, Khoshnavaz R, Emadzadeh M (2005). Determination of interleukin-6 and tumor necrosis factor-alpha concentrations in Iranian–Khorasanian patients with preeclampsia. BMC Pregnancy Childbirth.

[CR38] Wang L, Yang Z, Wang S, Wang S, Liu J (2011). Antioxidant and antibacterial activities of *Camptotheca acuminate* D. seed oil. Afr J Microbiol Res.

[CR39] Xu Q, Li YH, Lu XY (2007). Investigation on influencing factors of 5-HMF content in *Schisandra*. J Zhejiang Univ Sci B.

